# The effect of music on pain, anxiety and satisfaction during nonstress testing

**DOI:** 10.1111/jep.14106

**Published:** 2024-07-22

**Authors:** Handan Özcan, Suna Oral, Şehma Kuruay, Banu Müşerref Yilmaz

**Affiliations:** ^1^ Department of Midwifery University of Health Sciences Faculty of Health Sciences Istanbul Turkey; ^2^ Department of Midwifery Midwifery, University of Health Sciences Faculty of Health Sciences Istanbul Turkey; ^3^ Department of Gynecology and Obstetrics University of Health Sciences Zeynep Kamil Women's and Children's Research and Research Hospital Istanbul Turkey

**Keywords:** anxiety, music, nonstress testing, pain

## Abstract

**Objective:**

To determine the effect of instrumental music played during Nonstress Test (NST) on pain, anxiety and satisfaction.

**Methods:**

This research was planned as randomised controlled. The sample of the study consists of 64 pregnant women. In data collection, a survey form containing socio‐demographic characteristics, State‐Trait Anxiety Scale, Visual Analog Scale (VAS) and Evgeny Grinko‐Valse piece were used as music.

**Results:**

Among the groups where music was applied, satisfaction with the procedure was significantly high (*X*_*2*_: 12.666, p: 0.049). No significant difference was detected between anxiety and pain conditions (*p* > 0.05). The scale scores of the groups before and after the procedure were evaluated; Before the procedure, significant differences were detected between the groups in terms of trait anxiety and fear of pain. As a result of the evaluation made after the procedure, there are significant differences between state anxiety and fear of pain (*p* < 0.05). It was determined that music played during the NST procedure reduced state and trait anxiety. Satisfaction levels are also higher among the group that is listened to music.

**Conclusion:**

A successful pregnancy is important for the health of mother and baby. Reducing anxiety and stress, especially during the examinations, ensures that the process continues successfully. It is recommended that music played during pregnancy examinations and screening tests be used in clinics and during the procedure to increase satisfaction and reduce anxiety.

## 1 INTRODUCTION

Maintaining a healthy pregnancy is important for the health of the mother and baby in the postpartum period. Check‐ups and screening tests planned to be performed at regular intervals after the detection of pregnancy allows the problems that may occur in this process to be detected as early as possible and to detect high‐risk pregnancies. While prenatal screening is performed; Screening tests that can be performed as early as possible and have high reliability are preferred.^1^ One of the most commonly used methods during pregnancy is the Nonstress Test (NST). NST is a noninvasive foetal evaluation method. During this test, the baby's heart rate and movements and its relationship with uterine contraction, if any, are recorded and it is checked that the baby is growing healthily. It is also possible for women to experience emotions such as stress, pain and anxiety during NST.^2^

Music therapy is one of the effective methods of coping with stress. In addition to activating the areas of the brain related to perception, thinking, learning, speech, movement and body control, music reduces pain and anxiety levels.^3^ There is very evidence that music therapy are effective in reducing anxiety across a wide range of. Results from Cochrane systematic reviews show that music interventions have a moderate to large effect on patients with coronary heart disease,^4^ anxiety in cancer patients,^5^ and mechanical ventilation patients.^6^ Listening to music during situations of acute stress, such as before an examination or while awaiting a medical procedure or surgery, has also been found to be effective in reducing anticipatory anxiety.^7^ It has been stated that it positively affects people's quality of life by reducing the levels of other stress hormones such as serum dehydroepiandrosterone, epinephrine, and cortisol. The use of music during pregnancy, during, and after birth is increasing day by day.^3^ It has been reported that music therapy is an effective method used to reduce the stress and anxiety of pregnant women during pregnancy, birth, and the postpartum period. Music therapy, which is used to prevent negative obstetric outcomes caused by stress, especially in high‐risk pregnancies, is a technique with easy use, low cost, and easy applicability. It is suggested that midwives and nurses should encourage women to listen to relaxing music they like, in interventions and practices that take into account the personal characteristics of pregnant women.^8^, ^9^ This planned study aims to determine the effect of instrumental music played during the Nonstress Test (NST) on pain, anxiety, and satisfaction. The hypothesis of the study; music played during the NST procedure reduces the pain and anxiety of pregnant women and increases their satisfaction during the procedure.

## 2 METHODS

### 2.1 Type of research

**Figure 1:**
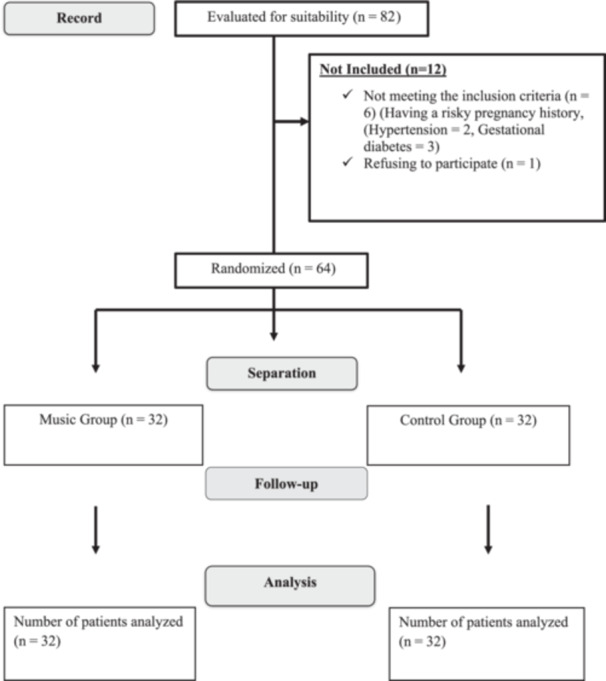
Consolidated Standards of Reporting Trials (CONSORT) diagram.

This study is a randomised controlled experimental study with pre‐test and posttest measurements, designed according to the Consolidated Standards of Reporting Trials (CONSORT) guideline (Figure 1).

### 2.2 Sample selection method

The research population consists of women who came to the pregnant outpatient clinics at X Training and Research Hospital in Istanbul for examination and had NST. Considering the literature information that each group should consist of at least 30 people in sample experimental studies, the power of the test was calculated with the G*Power 3.1 Programme. In our study, it was calculated that to reach a 90% power level with an effect size of 0.75 and a margin of error of 5%, the experimental and control groups should have at least 32 observations each, and the sample of the study was calculated as 64 pregnant women.

After the inclusion criteria for the study were determined, pregnant women were divided into experimental and control groups by simple randomisation method. In simple randomisation, participants to be assigned to groups were determined using a programme that generates random numbers (random.org).

*Criteria for inclusion in the study:* Applying to the clinic for NST procedure, aged 32–41. The inclusion criteria are that the woman is in her first week of gestation, wants to participate in the study voluntarily, and gives her consent.

Conditions to be met before NST procedure;

Having urinated just before the NST procedure

Having eaten at least 2 h before the NST procedure,

*Exclusion criteria for the study:* Experiencing any situation that causes the woman's anxiety to increase (such as mentioning a risky situation during the examination, developing a situation that disturbs her during the examination or waiting), having a multiple pregnancy, having a history of a risky pregnancy, being under control due to a risky situation. Exclusion criteria for the study include applying for a purposeful NST procedure, no uterine contractions as a result of NST, vital signs not being within normal limits, and a diagnosed cardiovascular disease in the foetus.

### 2.3 Data collection tools

In data collection, it was decided to use the Evgeny Grinko‐Valse work, which was determined as a result of a pilot study with a survey form containing socio‐demographic characteristics, State‐Trait Anxiety Scale, Visual Analog Scale (VAS), X model phone and Bluetooth speaker.

Music is an important way to trigger emotions and allow patients to express themselves verbally. Music therapy stimulates cognitive function, acts on levels of anxiety and depression, and therefore significantly improves autonomy in patients with psychiatric disorders. Listening to slow rhythm and relaxing music, especially piano music, has a relaxing effect in patients with anxiety and depressive disorders, as well as in individuals without psychiatric disorders. Guetin et al. In a study they conducted, it was reported that music therapy sessions were beneficial in anxiety and depression levels starting from the first session. Significant improvements were observed in anxiety and depression levels, and this situation was significantly maintained in subsequent sessions.^8^ For this study, Evgeny Grinko's Valse composition was chosen as slow rhythm piano music.

#### 2.3.1 Pre‐procedure pregnant information form

The pregnancy information form was prepared by the researcher in line with the literature; It includes questions about socio‐demographic characteristics such as age, gender, marital status, educational status, profession, obstetric history, and knowledge and experiences regarding NST. The last part of the survey consists of questions about music and information about the evaluation of pain status.

#### 2.3.2 Postprocedure pregnant information form

The pregnancy information form, prepared by the researchers in line with the literature, includes questions about the patient's pain and satisfaction after NST and consists of seven questions.

#### 2.3.3 State‐trait anxiety inventory

The scale consists of two parts.

1. State Anxiety Scale (SAS): It describes how an individual feels at a certain moment and condition.
2. Trait Anxiety Scale (TAS): It describes how the individual usually feels.

The State‐Trait Anxiety Inventory, developed by Spielberger et al. in 1970, was translated into Turkish by Öner and Le Compte (1985).^9^, ^10^ The scale consists of 40 items and has a 4‐point Likert feature. It is stated that the higher the score obtained from the scales, the higher the anxiety of the person. The Cronbach alpha coefficient of the scale is 0.87. In this study, the Cronbach alpha coefficient was determined as 0.796 before the procedure and 0.859 after the procedure.

#### 2.3.4 Fear of Pain Scale‐III

The Turkish validity and reliability study of the Fear of Pain Scale‐III (SCD), developed by McNeil and Rainwater in 1998, was conducted by Ünver and Turan.^11^ Fear of Pain Scale‐III is a 5‐point Likert‐type scale consisting of 30 items and three subscales. There are sub‐dimensions ‘Fear of Severe Pain’, ‘Fear of Mild Pain’ and ‘Fear of Medical Pain’. The lowest possible score in total is 30 and the highest score is 150. The lowest score that can be obtained for the sub‐dimensions is 10 and the highest score is 50. A high score on the scale indicates that the individual's fear of pain is also high. Cronbach's alpha reliability coefficient of the scale was found to be 0.938 for all items. In this study, Cronbach's alpha reliability coefficient was determined as 0.930 before the procedure and 0.950 after the procedure.

#### 2.3.5 Visual Analog Scale (VAS)

In the study, a 10 cm Visual Analog Scale (VAS) was used to evaluate pain and satisfaction status. The validity and reliability of the scale in Turkish was determined by Aydin et al.^12^

### 2.4 Collection of data

The patients in the intervention group were informed about music therapy, their consent was obtained and the necessary preliminary tests were performed. Starting from the woman's preparation stage until the NST procedure was completed, Evgeny Grinko‐Waltz music was played via a Bluetooth speaker. After the procedure, pain and satisfaction were scored using the Patient Information Form, Situational Anxiety Scale, and VAS.

Consent of the pregnant women in the control group was obtained, and post‐tests were performed after the pre‐test and routine NST procedure. No intervention was made to the pregnant women during this procedure.

### 2.5 Ethical approval

To conduct the research, the necessary approval was received from the Ministry of Health and the Scientific Ethics Committee of Zeynep Kamil General and Children's Diseases Training and Research Hospital. (Number: E‐15916306‐604.01.01‐213166037; Date: 09.04.2023).

### 2.6 Statistical method(s)

The data obtained from the study were analysed with the SPSS for Windows (version 20.0, Statistical Package for Social Sciences) programme. The distribution of the data was examined according to Skewness/Kurtosis values and it was found to be in accordance with the normal distribution. Statistics of continuous variables in the study were given as mean, standard deviation, minimum, and maximum values. Independent two‐sample T test was used to compare independent groups. The Bonferroni test was used in post hoc analysis to identify the differences between groups. Paired Sample T‐test was applied to compare the pre‐test and posttest of dependent samples.

## 3 RESULTS

The average age of the pregnant women participating in the study is 29.70 ± 5.48 (min‐max = 21–42). 41.4% of the participants and 40% of their spouses have undergraduate and graduate education, 74.3% are housewives and 55.7% have income equal to their expenses. 11.4% are related to their spouses, 15.7% smoke, and 37.1% have unplanned pregnancies (Table 1).

**Table 1:** Some socio‐demographic characteristics of pregnant women.

Age (mean ± sd, min‐max): 29.70 ± 5.48 (min‐max = 21–42),					
The age of spouse (mean ± sd, min‐max): 33.22 ± 6.96 (min‐max = 24–48).					
Education level	n	%	Employment status	n	%
Primary‐secondary education	22	31.4	Primary‐secondary education	14	20.00
High school	19	27.1	High school	28	40.0
Undergraduate and above	29	41.4	Undergraduate and above	28	40.0
Total	70	100.0	Total	70	100.0

Occupational status	n	%	Relative status of the spouse	n	%
Housewife	52	74.3	Yes	8	11.4
Working	18	25.7	No	62	88.6
Total	70	100.0	Total	70	100.0

Income status	n	%	Smoking status	n	%
Income less than expenses	24	34.3	Yes	11	15.7
Income equal to expenses	39	55.7	No	56	80.0
Income more than expenses	7	10.0	I quit	3	4.3
Total	70	100.0		70	100.0

54.3% of the pregnant women participating in the study define themselves as stressed, 74.3% use coping methods in a stressful situation, and the most commonly used methods are telling others (23.1%), walking (21.2%), listening to music (15.4%) and It was determined that there were other methods (32.7%).

84.3% of the pregnant women had NST before, 72.9% had information about NST, 58.5% received information from medical personnel, and 17.1% experienced tension during the procedure.

When some characteristics of the groups were compared, such as age, education, spouse's education, whether the pregnancy was planned, and income status, no statistically significant differences were found (*p* > 0.005). There is a significant difference between the comparison of satisfaction with the NST application (according to VAS scoring). Satisfaction levels were significantly higher among the music‐applied groups (X2: 12.666, p: 0.049). No significant difference was detected between anxiety and pain conditions (*p* > 0.05).

The groups' pre‐procedure and postprocedure scale scores were evaluated; Before the procedure, significant differences were detected between the groups in terms of trait anxiety and fear of pain. As a result of the evaluation made after the procedure, there are significant differences between state anxiety and fear of pain (*p* < 0.05, Table 2). While there is a decrease in state anxiety, trait anxiety and fear of pain scores in the music group, the scores are the same in the control group.

**Table 2:** Comparison of the groups' pre‐procedure and postprocedure scale scores.

	Music group (N: 35)		Control group (N: 35)			
Pre‐procedure	Mean ± SD	Min‐max.	Mean ± SD	Min‐max.	U	*p*
State Anxiety Scale (SAS)	31.62 ± 10.42	20–73	30.77 ± 8.60	20–73	0.033	0.709
Trait Anxiety Scale (TAS)	43.54 ± 8.19	26–64	37.85 ± 9.08	20–64	0.479	**0.008**
Fear of Pain Scale (FPS)	77.54 ± 26.82	30–123	61.91 ± 25.66	30–123	0.150	**0.016**
**Post procedure**						
State Anxiety Scale (SAS)	25.62 ± 6.17	20–44	30.71 ± 8.09	20–51	5.436	**0.004**
Trait Anxiety Scale (TAS)	39.68 ± 7.09	26–52	37.54 ± 7.80	22–58	0.026	0.234
Fear of Pain Scale (FPS)	76.48 ± 25.43	30–128	61.02 ± 27.47	30–128	0.232	**0.017**

*Note*: Bold values indicate statistical significant at *p* < 0.05.

Abbreviation: U, Mann‐Whitney U test.

As a result of the evaluation of the pre‐test and posttest of the groups in the study; It was determined that there were highly significant differences, especially in state and trait anxiety situations (*p* < 0.05, Table 3).

**Table 3:** Evaluation of pre‐study and post‐study scale scores of women in the music and control groups.

Music group				
	n	Average ± SD	z	*p*
**SAS pre test**	35	31.62 ± 10.42	−3.843	**0.001**
**SAS post test**	35	25.62 ± 6.17		
**TAS pre test**	35	43.54 ± 8.19	−3.672	**0.001**
**TAS post test**	35	39.68 ± 7.09		
**FPS pre test**	35	77.54 ± 26.82	−0.206	0.837
**FPS post test**	35	76.48 ± 25.43		

**Control group**				
	n	Average ± SD	z	*p*
**SAS pre test**	35	30.77 ± 8.60	−0.272	0.786
**SAS post test**	35	30.71 ± 8.09		
**TAS pre test**	35	37.85 ± 9.08	−0.314	0.753
**TAS post test**	35	37.54 ± 7.80		
**FPS pre test**	35	61.91 ± 26.66	−0.243	0.808
**FPS post test**	35	61.02 ± 27.47		

*Note*: Bold values indicate statistical significant at *p* < 0.05.

Abbreviations: MENQOL, Menopause‐Specific Quality of Life Questionnaire; WHIIRS, Women Health Insomnia Rating Scale; z, Wilcoxon signed‐rank test.

## 4 DISCUSSION

Pregnancy is one of the most important events that a woman experiences throughout her life, and although it is a natural event, it also brings with it many physiological, psychological and social changes.^13^ When pregnancy is planned, the effects of undesirable health consequences on the woman, foetus, and newborn are reduced, and health risks for the mother and baby are reduced to a minimum level.^13^, ^14^ 40% of pregnancies around the world are unplanned, and 62% of these pregnancies end in miscarriage and 38% in birth.^14^ The pregnancy of 37.1% of the pregnant women participating in this study was unplanned and the result is similar to the literature.

Scientific studies reveal that stress has negative effects on human health. Stress exposure and affective problems of pregnant women cause negative consequences in the development and health of the mother and child.^15^, ^16^ Approximately half of the pregnant women participating in the study define themselves as stressed (54.3%) and 74.3% of them use methods to cope with stress. It is important to control stress and healthily maintain the process, especially during pregnancy. With this awareness, healthcare personnel should minimise stress and provide appropriate environments and conditions during the follow‐up, treatment, and interviews of pregnant women.

World Music Therapy Federation defines music therapy as; It defines it as the use of music and/or musical elements (sound, rhythm, melodies, or harmonies) to facilitate and enhance communication, relationships, learning, movement, expression, organisation, and other related therapeutic goals. Thus, it is emphasised that physical, emotional, mental, social, and cognitive needs can be resolved with music. It is suggested that music therapy can be used to increase the success of the process in medical interventions.^17^, ^18^ In this planned study, the effect of music played to pregnant women during NST, one of the screening tests routinely applied to pregnant women, was discussed. It was determined that the state and trait anxiety levels of the group that listened to music during the procedure decreased significantly. Studies in literature show that music; It reduces anxiety, fear of birth, and the risk of premature birth, increases foetal‐maternal bonding, ensures relaxation of muscles, increases endorphin secretion in the body, supports treatment as a complementary and alternative treatment for pregnant women experiencing pre‐eclampsia by lowering blood pressure, and helps the foetus become more active by reducing the maternal heart rate and respiratory rate. It is reported that it helps people live in a healthy and comfortable environment^19^, ^20^ and causes changes in the immune and endocrine systems and mind and body processes.^21^ In parallel with this study, in a meta‐analysis study in the literature where the effect of music therapy on pain and anxiety during birth was evaluated, it was reported that it had positive effects on pain intensity and anxiety during birth.^17^ It seems that music can be used as an alternative method to reduce anxiety during pregnancy and birth.

Additionally, in this study, it was determined that the music played to pregnant women during the NST procedure also increased their satisfaction rates. In the study conducted by Sis Çelik and Atasever (2020), it was stated that the perceived stress levels were high in the study on stress factors and levels in 740 pregnant women who applied to the gynaecologist clinic at the hospital for prenatal control.^21^ It has been stated that pregnant women should find methods to cope with stress, reduce their anxiety, and organise training programmes to help them apply these methods. In this study, while there was a significant difference in the anxiety levels and satisfaction of pregnant women who listened to music during the NST procedure, there was no significant difference in their fear of pain before and after the procedure. It is observed that NST is not a painful procedure and pregnant women's anxiety increases during the NST procedure. To continue the process successfully, it seems that music therapy can be applied easily due to its ease of use, low cost, and noninvasive procedure.

## 5 CONCLUSION

It was determined that music increased satisfaction and comfort and reduced stress during NST, which is one of the procedures applied in the diagnosis and follow‐up of pregnancy. The use of music, which is one of the most suitable methods in terms of low cost and ease of use, is recommended to reduce the mother's stress and increase comfort during the procedures performed during pregnancy.

## CONFLICT OF INTEREST STATEMENT

The authors declare no conflict of interest.

## ETHICS STATEMENT

Informed consent was obtained from all individual participants included in the study. To conduct the research, the necessary approval was received from the Ministry of Health and the Scientific Ethics Committee of Zeynep Kamil General and Children's Diseases Training and Research Hospital. (Number: E‐15916306‐604.01.01‐213166037; Date: 09.04.2023).

## Data Availability

The data that support the findings of this study are available from the corresponding author upon reasonable request. The datasets generated during and analysed during the current research are available from the corresponding author on reasonable request.
